# Regulation and Methylation of Tumor Suppressor MiR-124 by Androgen Receptor in Prostate Cancer Cells

**DOI:** 10.1371/journal.pone.0116197

**Published:** 2015-04-10

**Authors:** Mingliang Chu, Yunli Chang, Yanjing Guo, Naitao Wang, Jian Cui, Wei-Qiang Gao

**Affiliations:** 1 State Key Laboratory of Oncogenes and Related Genes, Ren Ji-Med X Clinical Stem Cell Research Center, Ren Ji Hospital, School of Medicine, Shanghai Jiao Tong University, Med X Research Institute, Shanghai 200127, China; 2 Department of Pathology, Guizhou Provincial People's Hospital, The Affiliated People's Hospital of Guiyang Medical University, Guiyang 550002, China; 3 School of Biomedical Engineering & Med-X Research Institute, Shanghai Jiao Tong University, Shanghai 200030, China; Innsbruck Medical University, AUSTRIA

## Abstract

Prostate cancer (PCa) is the most frequently diagnosed cancer for men in the developed world. Androgen receptor signaling pathway plays an important role in prostate cancer progression. Recent studies show that microRNA miR-124 exerts a tumor suppressive function in prostate cancer. However, the relationship between AR and miR-124 is unclear. In the present study, we found a negative feedback loop between AR and miR-124 expression. On one hand, miR-124 was a positively regulated target gene of the AR, on the other hand, overexpression of miR-124 inhibited the expression of AR. In addition, we found that miR-124-2 and miR-124-3 promoters were hypermethylated in AR-negative PCa cells. Furthermore, overexpression of miR-124 inhibited proliferation rates and invasiveness capacity of PCa cells *in vitro*, and suppressed xenograft tumor growth *in vivo*. Taken together, our results support a negative feedback loop between AR and miR-124 expression. Methylation of miR-124-2 and miR-124-3 may serve as a biomarker for AR-negative PCa cells, and overexpression of miR-124 might be of potential therapeutic value for the treatment of PCa.

## Introduction

MicroRNAs (miR) are endogenous, single-stranded small non-coding RNAs (approximately 20–22 nucleotides) which usually cause gene silencing by reducing mRNA stability and/or translation [1]. Their aberrant expression has been found to be associated with several types of cancers [2–4]. MiR-124 is a highly conserved microRNA that plays a tumor suppressor role in various kinds of cancers [5–9]. The mature sequence of miR-124 is processed from three precursor variants sequences, which are located at chromosomes 8p23.1 (miR-124-1), 8q12.3 (miR-124-2) and 20q13.33 (miR-124-3), all of which contain CpG islands in their promoter region [9].

CpG islands are genomic regions that contain a high frequency of CpG sites and typically located near transcription start sites [10]. The methylation of CpGs in these regions is believed to lead to transcriptional silencing of their downstream genes [10]. Recent studies have shown that methylation is a reason for the low expression of miR-124 in cervical cancer [9], acute lymphoblastic leukemia [5], hepatocellular carcinoma [6], and pancreatic cancer [7]. However, Raynal et al. [11] reported that DNA methylation does not stably block gene expression since histone deacetylase inhibitors can reactivate gene expression without affecting methylation status [11]. But the patterns of methylation can serve as epigenetic markers for long-term maintenance of gene silencing [11]. Due to its specificity and stability in human samples, DNA methylation profiles may be as a useful biomarker in cancer screening [12, 13]. Consistent with this notion, recent studies have indicated that aberrant DNA methylation of miR-124 variants provide a valuable biomarker for cervical cancer [9], bladder cancer [14] and clear cell renal cell carcinoma [15].

Prostate cancer (PCa) is the most common malignancy among elderly men in the developed world with increasing rates in the developing countries [16]. Androgen receptor (AR) signaling pathway is very important in prostate cancer [17], which regulates many other genes involved in tumor progression or tumor suppression [18]. In addition, it has been shown that AR is correlated with the methylation status of specific microRNAs in PCa cells [19]. However, the mechanisms underlying regulation and methylation of miR-124 are still unclear.

The present study is aimed to study the regulation mechanism of miR-124, the methylation status of miR-124 and the anti-tumor function of miR-124 in PCa cells. The results of our experiment revealed that AR signaling promoted expression of miR124, while miR124 suppressed AR expression. There was a negative feedback loop between miR124 and AR expression. In addition, DNA methylation analysis showed that high methylation of miR124-2 and miR-124-3 occurred in AR-negative PCa cells, suggesting that this phenotype might be used as a biomarker for AR-negative PCa cells. Furthermore, overexpression of miR-124 showed anti-tumor activity *in vitro* and *in vivo*, suggesting a potential therapeutic value of miR-124 for the treatment of PCa.

## Materials and Methods

### Prostate cancer cell cultures and dihydrotestosterone treatment

LNCaP, 22Rv1, DU145 and PC-3 cell lines were obtained from the American Type Culture Collection (ATCC, Manassas, VA, USA). C4-2 cells, a subline of LNCaP cells, were obtained from UroCore (Oklahoma City, OK, USA). PC3/AR cells are the PC3 cells that stably overexpress the wild-type AR gene [20]; PC3/neo cells are a control cell line. Cells were maintained according to the manufacturer and providers' protocols. For Dihydrotestosterone (DHT, Sigma-Aldrich Co., St Louis, MO, USA) treatment, the medium containing 10% fetal bovine serum (Hyclone, Logan, UT, USA) was replaced by 10% charcoal-stripped bovine calf serum (Bioind, Israel) when LNCaP cells were 60%-70% confluent, and then 0, 5 and 10 nM DHT were added respectively and cells were further cultured for 12 hours.

### Plasmids, lentiviral production and infection

A 21mer sequence (5-GGTGTCACTATGGAGCTCTCA-3) was designed to generate the lentiviral vector of AR short hairpin RNA (shRNA) [21], and the vector construct, lentiviral production and infection were done according to previous description [19]. The lentiviral plenti-CMV-miR-124 vector was constructed to stably overexpressing mature sequence of miR-124. The vector of pLenti CMV/TO Puro empty (Addgene plasmid #17482 [22]) was purchased from Addgene. The genomic segment of miR-124 -2 sequences was amplified using primers miR-124-2-F: CCTGGATCCGCTGTAAATGGCATGGAGATAT and miR-124-2-R: GCGTCTAGAGCGGCTGTAATGGAAAAGTAG; and subcloned into BamH1 and Xbal sites of the pLenti CMV/TO Puro empty vector [23]. Lentivirus production and transduction were done according to a previous method [23].

### Real-Time PCR

Total RNAs were extracted using the RNeasy Plus Mini kit (Qiagen, Valencia, CA, USA) from various PCa cells. Reverse transcription was performed using the PrimeScript RT reagent kit (TaKaRa, Dalian, China) and the TaqMan MicroRNA Reverse Transcription Kit (Applied Biosystems, Foster City, CA, USA) according to the manufacturer’s instructions. Relative quantities of miR-124 (using TaqMan microRNA Assays, Applied Biosystems) and AR (using the SYBR Green Realtime PCR Master Mix, TOYOBO, Osaka, Japan) were performed with a 7500 Real Time PCR System (Applied Biosystems). Gene expression was normalized by the endogenous control RNU44 (miR-124) or β-actin (AR). The Ct values were calculated using the ΔΔCt method. The following primers were used: AR forward CCAGGGACCATGTTTTGCC, reverse CGAAGACGACAAGATGGACAA; β-actin forward CATGTACGTTGCTATCCAGGC, reverse CTCCTTAATGTCACGCACGAT.

### Sodium bisulfite modification and sequencing

The methylation status of each miR-124 variants was determined by Bisulfite-Sequencing PCR (BSP) after bisulfite conversion of cells using the EZ DNA methylation-direct kit (Zymo Research Corporation, Irvine, CA, USA) following standard protocols. BSP Primers were designed using the Methyl Primer Express v1.0 software (Applied Biosystems, Foster City, CA, USA) (Table 1). The PCR products were ligated into the pMD19-T Simple Vector (TaKaRa Biotechnology Co., Ltd., Dalian, China). Colonies (n = 10) were selected per PCa cell line and sequenced by Shanghai Shenggong Biotech (Shanghai, China).

**Table 1:** Bisulfite-Sequencing PCR Primers.

**Gene**	**Primers(5'-3')**	**Size (bp)**	**Annealing (°C)**
miR-124-1	F:ATTGTTTTTTTGGTTTTAGGGA	337	58
	R:TAACCCCTCATCAACTTTATCA		
miR-124-2	F:TGTTGTAAATGGTATGGAGATATATGT	253	58
	R:AAAACAAAACCTCTAATCTTAATCC		
miR-124-3	F:GGGAGAAGTGTGGGTTTTTT	244	58
	R:CCTTAATTATATAAACATTAAATCAAAATC		

F: forward; R: reverse; bp: base pairs.

### Cell proliferation assays

Cell proliferation was measured using a cell counting kit (CCK-8; Dojindo, Kumamoto, Japan) assay. Cells were plated in 96-well plates with 5,000 cells per well and examined at 48, 72, 96 hours after plating (n = 12). CCK-8 (10 μL) was added to each well and the cells were incubated for an additional 2 hours. The absorbance was measured with a microplate reader (BioTek, USA) at wavelengths of 450 nm.

### Migration assays

Costar Transwell Migration Plates with 8μm pore size (#3422, Corning, USA) were used. The upper chamber were seeded with 1 x 10^5^ cells in serum-free media and 20% FBS was used as the attractant in the lower chamber. After 24 hours of culturing, the culture inserts were removed and washed with phosphate buffer saline (PBS) several times to get rid of unattached cells. All the remaining cells on the upper side were scraped with a cotton swab. Migrated cells on the lower side of the insert were fixed in 4% paraformaldehyde for 15 minutes, washed with PBS twice, and stained with 0.1% crystal violet for 10 minutes. For each insert, 5 randomly selected regions of the filter were counted under a light microscope. Each of the experiment was repeated three times.

### *In vivo* xenograft assays

Six weeks old male BALB/C nude mice (SLAC, Shanghai, China) were randomly grouped and subcutaneously injected with 4x 10^6^ LNCaP-control or LNCaP-miR-124 cells mixed with an equal volume of matrigel (BD Biosciences, Bedford, MA, USA). Each group had at least five mice. Tumors were measured with a caliper every 10 days from 2 weeks after inoculation and the volume was calculated as π/6 × length × width^2^. Xenograft tumors were excised and weighed at sacrifice on 34 day post-tumor cell injection. All mouse experiments were approved by the Renji hospital animal care and use committee.

### Statistical analysis

Data were analyzed using the SPSS 13.0 softwares (SPSS Inc., Chicago, Ill., USA) and Prism GraphPad 5 (GraphPad Software, La Jolla, Calif., USA). Statistical analysis was performed using the Student’s t-test. Data are presented as the means ± SEM from at least three independent experiments. Probability values less than 0.05 were considered significant.

## Results

### A negative feedback loop between miR-124 and AR expression

**Fig 1:**
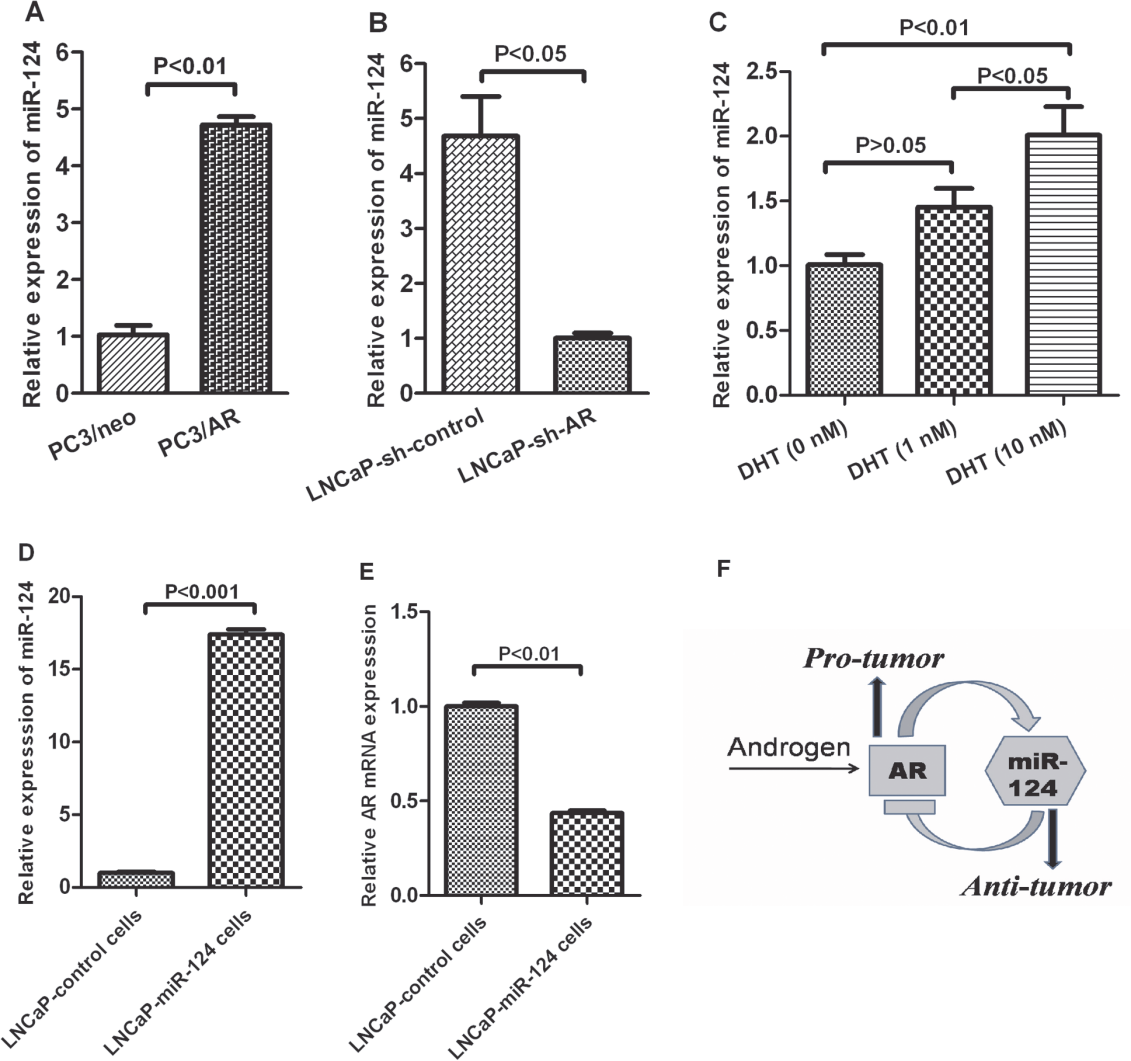
A Negative Feedback Loop Between MiR124 and AR Expression. (A) PC3/AR cells are a stable cell line overexpressing human AR cDNA; PC3/neo cells are used as a control. (B) LNCaP-sh-AR cells are AR-knockdown cells, in which LNCaP cells were infected with lentivious AR shRNA; LNCaP-sh-control cells are used as a control. (C) 0 nM, 1 nM and 10 nM DHT were added to LNCaP cells and cultured for 12 hour. (D) LNCaP cells were infected with a control lentivirus (LNCaP-control cells) or plenti-CMV-mir-124 lentivirus (LNCaP-miR-124 cells). Relative expression of miR-124 in LNCaP cells was determined by qRT-PCR and corrected to RUN44 levels. Values mean fold-changes normalized to (A) PC3/neo cells; (B) LNCaP-sh-AR cell; (C) LNCaP cells (0 nM DHT) and (D) LNCaP-control cells. (E) Relative expression of AR was determined by qRT-PCR and normalized to β-actin levels. Values mean fold-changes normalized to LNCaP-control cells. (F) A schematic diagram of the pathway described in the study. Data are shown as the means ± SEM from 3 independent experiments, each of which were performed in triplicates

To determine whether AR regulates expression of miR-124 in PCa cell lines, AR overexpression and AR knockdown experiments were performed in PC3 cells (vs. PC3/AR) and LNCaP cells (vs. LNCaP-sh-AR), respectively. As shown in Fig. 1, while overexpression of the AR in PC3 cells enhanced expression level of miR-124 (Fig. 1A), knockdown of AR in LNCaP cells reduced the expression level of miR-124 (Fig. 1B). Furthermore, we examined if miR-124 is an androgen responsive microRNA. LNCaP cells, androgen-responsive PCa cells, were plated in androgen-free medium and treated with DHT for 0 nM, 1 nM and 10 nM, respectively. After 12 hour, the RNAs were extracted and real time PCR was analyzed. These experiments revealed an increase for the expression amounts levels of miR-124 gene in LNCaP cells treated with DHT (10 nM) treatment (Fig. 1C). Above results suggesting that AR is closely positively correlated with the expression of miR-124. However, it is not clear whether there exists a feedback influence between miR-124 and AR. To study this possibility, LNCaP cells were infected with a control lentivirus (LNCaP-control cells) or plenti-CMV-mir-124 lentivirus (LNCaP-miR-124 cells) which expresses high levels of miR-124 (Fig. 1D). Expression level of AR was then analyzed by qRT-PCR. As shown in Fig. 1E, overexpression of miR-124 significantly inhibited AR mRNA level, which is consistent with a previous study reporting that treatment with miR-124 resulted in a reduction of AR protein in LNCaP cells [8]. Taken together, these results suggested a negative feedback loop between miR-124 and AR expression (Fig. 1F).

### Methylation analysis of miR-124 in PCa cells

**Fig 2:**
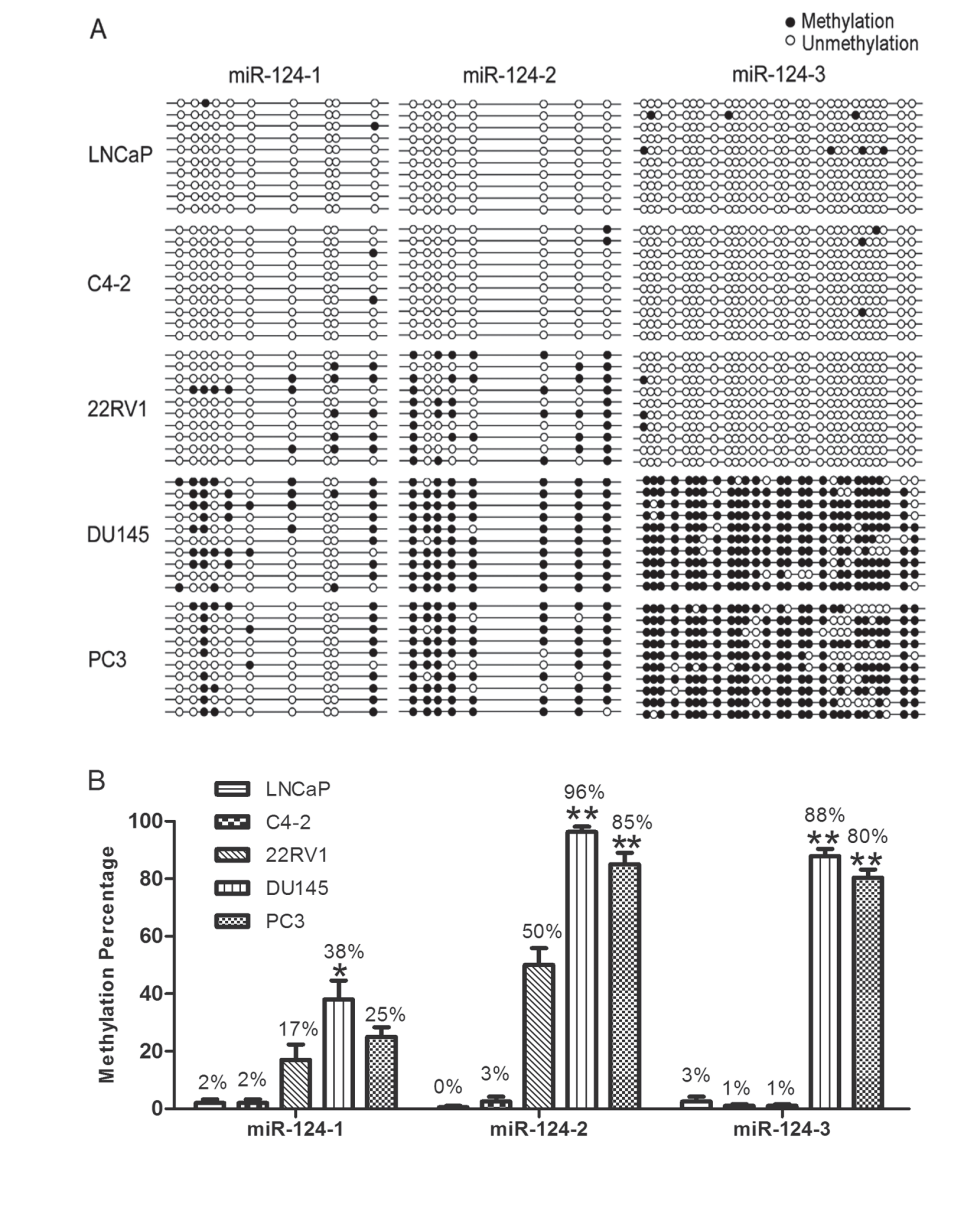
Methylation Status of MiR-124-1, MiR-124-2 and MiR-124-3 CpG Islands. Schematic summary of CpG sites in the miR-124-1, miR-124-2 and miR-124-3 promoter regions. (A) Methylation analysis was done in 10 clones from each cell line. Each row of circles represents a single clone, and each circle represents a single DNA methylated or demethylated site. (B) The methylation percentages of 10 clones from each of the cell lines are summarized in the bar graph. Data are shown as the means ± SEM. Asterisks indicate P<0.05, double asterisks indicate P<0.001.

The mature miR-124 contains 3 premature variants: miR-124-1, miR-124-2 and miR-124-3 (20q13.33). Bisulfite sequencing PCR (BSP) and gene sequencing were performed to analyze the DNA methylation levels of each of the miR-124 variants. These analyses showed that methylation activities of miR-124-2 and miR-124-3 were higher in AR-negative PCa cells (85%-96%, 80%-88%; respectively) than in AR-positive PCa cells (0%-50%, 1%-3%; respectively) (Fig. 2). However, in both AR-negative and AR-positive PCa cells, the promoter region of miR-124-1 was methylated to only a limited degree (2%-38%, Fig. 2). In addition, miR-124-1 methylation was not significantly higher than the AR-positive cell in the AR-negative PCa cells (DU145, PC3) except for DU145 cells in which methylation was significantly higher than AR-positive cells (22RV1, C4-2 and LNCaP; p<0.05).

### MiR-124 overexpression suppresses LNCaP cells proliferation, migration and xenograft tumors growth

**Fig 3:**
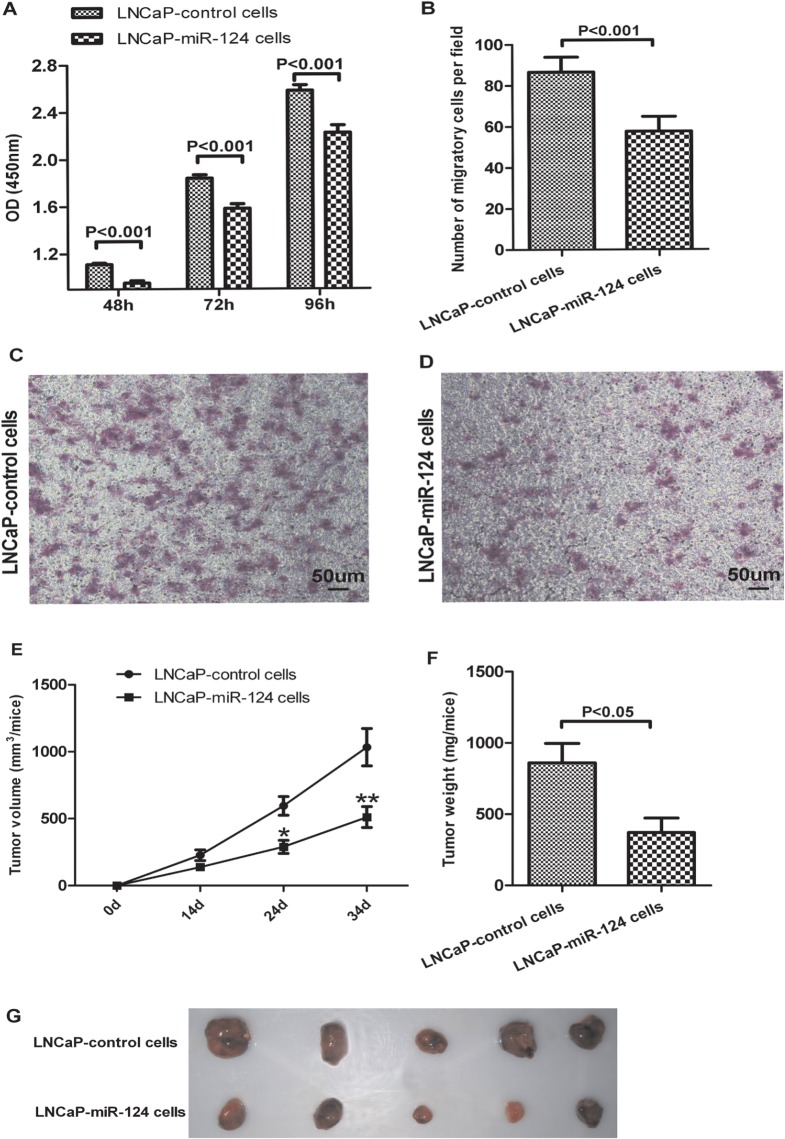
Overexpression of MiR-124 Suppresses LNCaP Cells Proliferation, Migration and Xenograft Tumors Growth. (A) LNCaP-control cells and LNCaP-miR-124 cells were plated in 96 wells and examined at 48, 72, 96 hours using CCK8 assay. Cell proliferation was assayed by the value of OD. Data are shown as the means ± SEM (n = 12). Group data (B) and representative images (C,D) are shown. (B) Number of migratory cells per field of LNCaP-control cells and LNCaP-miR-124 cells. Representative photomicrographs illustrating migration of LNCaP-control cells (C) and LNCaP-miR-124 cells (D). Five nude mice per group were injected subcutaneously with 4x10^6^ LNCaP-control cells or LNCaP-miR-124 cells. (E) The volume of xenograft tumors were measured every 10 days from 2 weeks after inoculation. (F) Tumor weights were measured at the time of sacrifice on 34 day after tumor cell injection. (G) Tumors were excised at the time of sacrifice on 34 day post tumor cell injection. Data are presented as means ± SEM. Asterisks indicate P<0.05, double asterisks indicate P<0.001.

Previous studies indicate that miR-124 plays a role as a tumor suppressor [5–9], so we wanted to investigator whether overexpression of miR-124 could inhibit PCa cells proliferation. To answer this question, LNCaP-control cells and LNCaP-miR-124 cells were plated in 96-well plates and examined at 48, 72, 96 hours after the initial plating (n = 12). Cell proliferation was measured by the absorbance (optical density, OD) of 450 nm wavelengths. Our results showed that overexpression of miR-124 significantly inhibited LNCaP cell proliferation at 48, 72 and 96 hours, respectively (Fig. 3A).

Furthermore, we determine whether overexpression of miR-124 suppresses PCa cell migration, the transwell chambers were used to evaluate the migratory effect of LNCaP-control cells and LNCaP-miR-124 cells. The transwell assays showed that overexpression of miR-124 significantly suppressed the migration of LNCaP cells (Fig. 3B,C,D). The observation that miR-124 inhibits LNCaP cells proliferation and migration prompted us to determine whether miR-124 overexpression can inhibit xenograft tumor growth of LNCaP cells *in vivo*. To answer this question, male nude mice were inoculated subcutaneously with LNCaP-control cells or LNCaP-miR-124 cells. We found that both groups of mice showed palpable xenograft tumors on 14 day after inoculation. However, the size of tumors were significantly smaller in LNCaP-miR-124 xenografts compared with the LNCaP-control xenografts after 24 days (Fig. 3E,G). In addition, we found that the weight of tumors were significantly lighter in LNCaP-miR-124 xenografts compared with the LNCaP-control xenografts at the time of sacrifice on 34 days (Fig. 3F,G). Collectively, overexpression of miR-124 not only inhibited LNCaP cell proliferation and migration *in vitro* but also significantly suppressed the growth of xenograft tumors *in vivo*.

## Discussion

In the present study, we have presented evidence that miR-124 is a androgen/AR responsive gene and overexpression of miR-124 inhibits AR expression and suppresses PCa cells proliferation, migration and xenograft tumor growth. These results suggest a negative feedback loop between AR and miR-124 expression, and miR-124 may be a promising therapeutic target for PCa.

Androgen/AR signaling is very crucial in the onset and progression of prostate carcinogenesis [24, 25]. Not only steroid hormones but also nonsteroidal compounds could activate AR signaling [26]. Besides, in "hormone-refractory prostate cancer", the AR signaling can be activated all the same [27]. In short, in androgen/AR signaling, AR plays a more important role than androgen. Therefore firstly we performed AR overexpression and knockdown experiments to verify whether AR signaling could influence the expression of miR-124. We found that the expression of miR-124 were positively correlated to AR. Secondly, treatment of LNCaP cells with DHT induced an increased expression of miR-124. Therefore AR positively regulates the expression of miR-124. It is noted that our results are not consistent with a previous report in which androgen analog R1881 was used and did not regulate miR-124 [8]. One explanation for this discrepancy might be due to the different research methods. Further studies are needed to clarify the inconsistent results.

The negative feedback loop that we observed between miR-124 and AR expression may play a role in the development of castration-resistant PCa (CRPC). As we know, androgen deprivation therapy that removes circulating androgens or blocks the AR is a standard of care treatment for prostate cancer treatment. Despite an initial effective response to therapy, almost all patients inevitably develop to CRPC. Although there has been a lot of research done, the mechanism is still unclear. Our results showed the androgen/AR signaling can positively influence the expression of miR-124, but the latter inhibits the expression of the former. We speculate that in normal prostate cell, the AR and miR-124 expressions are at a balance as proposed for that occurring between proto-oncogenes and tumor suppressors [28]. If the AR signaling is activated, the expression of down-stream miR-124 would be high, and inversely inhibits the AR signaling. However, the balance is disrupted in PCa, that is, the AR signaling can be further enhanced, when the expression of miR-124 is low [8].

Given that methylation plays a role in the regulation of gene expression, the present study has investigated whether the expression of miR-124 was associated with its methylation status. Mature sequence of miR-124 has three precursor variants, miR-124-1, miR-124-2 and miR-124-3, all of which were analyzed for the methylation status in our studies. Besides miR-124-1, the promoter regions of miR-124-2 and miR-124-3 are highly methylated in AR-negative PCa cells than AR-positive PCa cells (Fig. 2). When expression of miR-124 is examined in PCa cells, it does not shown consistency with the methylation status of PCa cells (S1 Fig.). This discordancy could be explained by a recent new theory suggesting that DNA methylation does not stably shut down gene expression but instead serves as a molecular marker for a long-term maintenance of gene silencing [11]. Due to DNA methylation represents a more chemically and biologically stable source of molecular diagnostic information than RNA or most proteins [29], it has great potential to be a useful informative biomarker in cancer screening [12, 13]. In this regard, our results suggest that aberrant hypermethylation of miR-124-2 and miR-124-3 may provide a helpful biomarker for AR-negative PCa cells.

It should be pointed out though overexpression of miR-124 can inhibit PCa carcinogenic process *in vitro* and *in vivo*, the knockdown of miR-124 in LNCaP cells does not change the carcinogenic status (data not shown). One explanation could be that the mature sequences of miR-124 have three precursor variants sequences, and it is difficult to profoundly downregulate the expression. However, further studies are needed to clarify this point.

Taken together, our results provide new insights into the regulating mechanism of tumor suppressor miR-124 and different methylation status of miR-124 variants might be used as a potentially useful biomarker for PCa cells.

## Supporting Information

S1 Fig Expression of MiR-124 in PCa Cells. Relative expression of miR-124 in PCa cell lines was determined by qRT-PCR and corrected to RUN44 levels. Values mean fold-changes normalized to LNCaP cells. Data are shown as the means ± from 3 separate experiments, each of which was performed in triplicates.
(DOC)
