# Supplementary material for: Regulation and Methylation of Tumor Suppressor MiR-124 by Androgen Receptor in Prostate Cancer Cells
Source: PLoS One. 2015 Apr 10;10(4):e0116197. doi: 10.1371/journal.pone.0116197 (PMC4393226; doi:10.1371/journal.pone.0116197)
Supplement: S1 Fig — Relative expression of miR-124 in PCa cell lines was determined by qRT-PCR and corrected to RUN44 levels. Values mean fold-changes normalized to LNCaP cells. Data are shown as the means ± from 3 separate experiments, each of which was performed in triplicates. (DOC) [file pone.0116197.s001.doc]

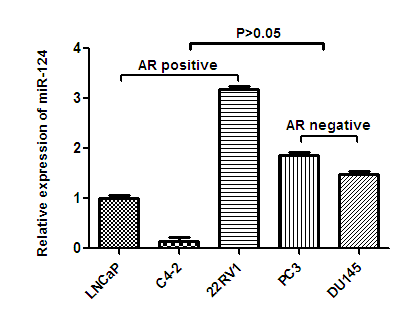


**Figure S1. Expression of MiR-124 in PCa Cells.**

Relative expression of miR-124 in PCa cell lines was determined by qRT-PCR and corrected to RUN44 levels. Values mean fold-changes normalized to LNCaP cells. Data are shown as the means ± from 3 separate experiments, each of which was performed in triplicates.
